# Joint FAO/IAEA Coordinated Research Project on “Exploring genetic, molecular, mechanical and behavioural methods of sex separation in mosquitoes” – an introduction

**DOI:** 10.1186/s13071-018-3206-9

**Published:** 2018-12-24

**Authors:** Kostas Bourtzis, Zhijian Jake Tu

**Affiliations:** 1 Insect Pest Control Laboratory, Joint FAO/IAEA Programme of Nuclear Techniques in Food and Agriculture, Vienna, Austria; 2 Department of Biochemistry, Virginia Tech, Blacksburg, VA 24061 USA

*Aedes* and *Anopheles* mosquitoes are major vectors of human diseases such as malaria, dengue, chikungunya, Zika and yellow fever. There is a lack of efficient drugs and vaccine for most of these vector-borne infectious diseases and traditional mosquito control methods, mainly based on larval source reduction and insecticides are inefficient. Therefore, new or complementary techniques, such as novel genetic strategies, are urgently needed to combat major mosquito vector species. There are three major types of genetic strategies to achieve vector population suppression: the sterile insect technique (SIT), the incompatible insect technique (IIT), and transgenic approaches. These techniques all rely on male-only releases as female mosquitoes bite and transmit pathogens. However, the lack of efficient and robust sex separation methods presents a major bottleneck to the successful application of these techniques as the currently available sex separation methods are inadequate for safe and bio-secure male-only releases in large scale operational programs [1, 2].

Five years ago, a coordinated research project (CRP) entitled “Exploring genetic molecular, mechanical and behavioural methods of sex separation in mosquitoes” was initiated under the auspices of the Joint Division of Nuclear Techniques in Food and Agriculture of the Food and Agriculture Organization (FAO) and the International Atomic Energy Agency (IAEA). Kostas Bourtzis, a member of the Insect Pest Control Subprogramme of the FAO/IAEA, coordinated the project which included 19 scientists with a broad range of expertise from 14 countries, to explore classical genetic, molecular, mechanical, behavioural, developmental and symbiont-based approaches for sex separation in mosquitoes. In the frame of this 5-year CRP project, four meetings were organized during which the participating scientists presented their findings and achievements as well as critically discussed and coordinated their research. This special issue presents, in the form of original and/or review papers, recent achievements in the field of mosquito sex separation, together with a perspective paper which discusses potential future steps.

Several review articles present the progress and challenges for the development of genetic sexing strains in *Aedes* and *Anopheles* mosquitoes by classical genetics and/or molecular-based approaches. Bernardini et al. [3] review the significant progress achieved during the last few years towards the development of genetic sexing strains using transgenic technologies for the control of *Anopheles gambiae*, which is a major vector of human malaria. Mashatola et al. [4] discuss various sex separation strategies that have been or are currently explored to develop a robust sex separation method which would allow SIT applications against *An. arabiensis* including the exploitation of sex-specific behavioural and developmental differences as well as genetic-based approaches such as genetic sexing strains. Haecker & Schetelig [5] present the pros and cons of using transposable elements, site-specific recombination, and genome editing methods in the context of developing genetic sexing strains for mosquito vector control. Araujo et al. [6] report on the need to deeply understand the mechanisms of sex determination in *Aedes* mosquitoes, which would allow the exploitation of the sex determination pathways to construct male-only strains.

Ndo et al. [7] report the isolation, establishment and the characterization of a temperature-sensitive lethal (tsl) mutant strain in *An. arabiensis* and describe its potential use for the development of a genetic sexing strain (GSS) similar to the one currently used worldwide for SIT applications against the major agricultural pest, the Mediterranean fruit fly *Ceratitis capitata* [8, 9]. Krzywinska & Krzywinski [10] show that the ectopic expression of the sex determining gene Yob largely results in female lethality or sterility while males remain viable and fertile. These data suggest that it is possible to establish *An. gambiae* transgenic genetic sexing strains with a conditional male-only phenotype. Smidler et al. [11] investigate the promoter of the major mating plug protein Plugin and use it to express fluorescence markers in *An. gambiae.* Successful insemination by males of these transgenic lines can be detected up to eight hours after copulation by monitoring the deposition of the florescent markers in mated females, thus facilitating the assessment of male mating competitiveness. Using RNAseq data, Wu et al. [12] identify pure early zygotic genes (pEZGs) whose promoter or regulatory sequences could be used to drive female-specific lethality to achieve sex separation and male-only releases.

As regards *Aedes* species, Gomulski et al. [13] identify the male-specific chromosome 1 of *Ae. albopictus* and characterize the putative male-determining gene Nix which paves the way for unravelling the sex determination pathway in this species as well as the development of novel genetic control strategies against this mosquito vector species. In addition, Hu & Tu [14] provide evidence that the promoter of the endogenous early zygotic gene KLC2 is active at the syncytial blastoderm and early cellular blastoderm as well as in male testes suggesting that it could be harnessed for the development of genetic sexing strategies in *Aedes aegypti*.

Bellini et al. [15] report that, although the selection for enhanced dimorphism in pupal size is challenging in *Aedes albopictus*, it is possible to select strains for enhanced protandry which may result to robust and efficient sex separation approaches is support of sterile insect technique applications. Interestingly, Zacarés et al. [16] report on the development of an automated pupal size estimator which was used to show that enhanced sexual size dimorphism (SSD)-based sex sorting methods can result to highly efficient sex separation, and at the same satisfactory male pupae recovery, in *Aedes aegypti*, *Ae. albopictus* and *Ae. polynesiensis*. A significant breakthrough is presented by Lebon et al. [17] who constructed the first genetic sexing strain (GSS) in *Ae. albopictus*, namely Tikok, based on the *rdl* gene conferring dieldrin resistance. Even though this GSS is based on an insecticide resistance gene, this achievement opens the door for large-scale sex separation critical for population suppression applications.

Using laboratory data and simulations, Moretti et al. [18] suggest that it is possible, under certain conditions, to apply in the field a *Wolbachia* bidirectional cytoplasmic incompatibility-based suppression strategy which will rely on the release of incompatible males together with a small percentage of contaminant females. However, the risk of such release strategies to end up in population replacement instead of population suppression would still exist [19, 20]. In the absence of efficient and robust methods for sex separation, and to avoid the release of even a small number of fertile and potentially pathogen transmitting females, the combination of sterile insect technique and incompatible insect technique (SIT/IIT), which combines irradiation with *Wolbachia* symbiosis, was suggested [19, 20]. This integrated approach was first developed in *Aedes albopictus* and tested under laboratory conditions [21–23].

In this special issue, Kittayapong et al. [24] discuss that, during a small scale *Ae. aegypti* suppression trial of combined SIT/IIT approach in Thailand, and due to the inefficiency of the mechanical approach based on glass larval-pupal separators, there was an accidental release of a small number of females. However, the released females were completely sterile due to the integration of irradiation, so there was no genetic footprint in the environment. In addition, due to the presence of *Wolbachia* and their short life span, the irradiated *Wolbachia*-infected females are unable to transmit any pathogens.

As an epilogue in this special issue, Papathanos et al. [25] highlight in a perspective manuscript the remaining bottlenecks and challenges facing the development of suitable sex separation approaches for the deployment of mosquito genetic population control strategies. They also suggest future directions for the development of efficient and robust sex separation methods, ideally based on genetic sexing strains, for *Anopheles* and *Aedes* mosquitoes.

It is our hope, as guest editors, that this issue of the Parasites & Vectors presents the current knowledge in the field of mosquito sex separation. We also hope that it will help to the design and support of future research activities for the development of efficient, robust and cost-effective sex separation methods which are urgently needed for large scale deployment of mosquito population control strategies.
